# Prediction of long noncoding RNA functions with co-expression network in esophageal squamous cell carcinoma

**DOI:** 10.1186/s12885-015-1179-z

**Published:** 2015-03-24

**Authors:** Yibin Hao, Wei Wu, Fachun Shi, Rodrigo JS Dalmolin, Ming Yan, Fu Tian, Xiaobing Chen, Guoyong Chen, Wei Cao

**Affiliations:** 1 Zhengzhou Central Hospital, Affiliated to Zhengzhou University, China, 195 Tongbai Road, Zhengzhou, 450007 PR China; 2 Department of Pathology and Experimental Medicine, University of Calgary, Calgary, AB Canada; 3 Science and Education Department, Health Bureau of ZhengZhou, Zhengzhou, China; 4 Department of Biochemistry, Bioscience Center and Institute of Tropical Medicine of Rio Grande do Norte, Federal University of Rio Grande do Norte, Natal, Rio Grande do Norte Brazil; 5 Medical School, Zhengzhou University, Zhengzhou, Henan China

**Keywords:** Long noncoding RNA, lncRNA, Co-expression network, Esophageal cancer, ESCC, ESCCAL-1, Cancer initiatome

## Abstract

**Background:**

Long non-coding RNAs (lncRNAs) are pervasively transcribed in the genome. They have important regulatory functions in chromatin remodeling and gene expression. Dysregulated lncRNAs have been studied in cancers, but their role in esophageal squamous cell carcinoma (ESCC) remains largely unknown. We have conducted lncRNA expression screening and a genome-wide analysis of lncRNA and coding gene expression on primary tumor and adjacent normal tissue from four ESCC patients, tend to understand the functionality of lncRNAs in carcinogenesis of esopheagus in combination with experimental and bioinformatics approach.

**Methods:**

LncRNA array was used for coding and non-coding RNA expression. R program and Bioconductor packages (limma and RedeR) were used for differential expression and co-expression network analysis, followed by independent confirmation and functional studies of inferred onco-lncRNA ESCCAL-1 using quantitative real time polymerase chain reaction, small interfering RNA-mediated knockdown, apoptosis and invasion assays in vitro.

**Results:**

The global coding and lncRNA gene expression pattern is able to distinguish ESCC from adjacent normal tissue. The co-expression network from differentially expressed coding and lncRNA genes in ESCC was constructed, and the lncRNA function may be inferred from the co-expression network. LncRNA ESCCAL-1 is such an example as a predicted novel onco-lncRNA, and it is overexpressed in 65% of an independent ESCC patient cohort (n = 26). More over, knockdown of ESCCAL-1 expression increases esophageal cancer cell apoptosis and reduces the invasion *in vitro*.

**Conclusion:**

Our study uncovered the landscape of ESCC-associated lncRNAs. The systematic analysis of coding and lncRNAs co-expression network increases our understanding of lncRNAs in biological network. ESCCAL-1 is a novel putative onco-lncRNA in esophageal cancer development.

**Electronic supplementary material:**

The online version of this article (doi:10.1186/s12885-015-1179-z) contains supplementary material, which is available to authorized users.

## Background

The human genome contains many thousands of long noncoding RNAs (lncRNAs), which are endogenous RNA transcripts with more than 200 nucleotides, and they lack protein coding potential [1]. LncRNAs are generally expressed at a lower level than protein-coding genes, they display more tissue-specific and cell-specific expression patterns [2]. A growing body of evidence has demonstrated that lncRNAs participate in numerous cellular processes ranging from embryonic stem cell pluripotency, cell-cycle regulation, and diseases, such as cancer [3]. An emerging theme of lncRNAs-mediated gene regulatory network is the interaction with ribonucleotprotein complexes and post-transcriptional modification of coding genes. The regulatory mechanisms are exemplified by X inactive specific transcript (Xist)-mediated X chromosomal inactivation [4] and HOX transcript antisense RNA (HOTAIR)-medicated *HOX D* cluster in Chr 2 [5]. With the advent of high-throughput microarray and DNA sequencing technologies, about 73,372 lncRNAs have been annotated in the mammalian genome [6]. However, only a fraction of the lncRNAs’ functions have been characterized experimentally. Hence, prediction of lncRNAs’ functions with multiple model systems provides guideline for further experimental investigations [7,8].

Oesophageal cancer (EC) is the eighth most common cancer worldwide, with an estimated 456,000 new cases in 2012 (3.2% of all cancer), and the sixth most common cause of death from cancer with an estimated 400,000 deaths (4.9% of the total) [9]. EC comprises of two different histopathological forms: esophageal adenocarcinoma (EAC) and esophageal squamous cell carcinoma (ESCC). Despite the advances in contemporary treatment, the outcome of EC remains looming. The underlying molecular mechanism of these two forms are distinct. Recent high-throughput cancer genome sequencing revealed a handful of common known somatic gene mutations (TP53, CDKN2A, SMAD4, ARID1A and PIK3CA) and novel somatic gene mutations including chromatin modifying factors in EAC [10], and some previously undescribed gene mutations (ADAM29 and FAM135B) were detected in ESCC [11]. While most researches on esophageal cancer still focus on 2% of coding genes in the genome, lncRNA biology opens the door to understand more about the cancer initiatome which is the collective information of cellular malignant transformation [12].

The role of lncRNAs in cancer has drawn great attention in recent years. Dysregulated lncRNAs in different cancer suggest that lncRNAs are an enigmatic component of the whole transcriptome, which may involve in tumorigenesis, invasion and metastasis [13]. Detection of aberrant expression of lncRNAs in various tissue origin of cancers could serve as novel biomarkers for cancer diagnosis and prognosis. For example, the cancer-related lncRNA, MALAT-1 (Metastasis-Associated in Lung Adenocarcinoma Transcript 1), was identified by subtractive hybridization during screening for early non-small cell lung cancer with metastasis. Elevated MALAT-1 expression was highly predictive of poor prognosis and shortened survival time in early stage lung cancer [14]. Up-expression of HOTAIR lncRNA was found in several solid tumors [15-19] in association with cancer metastasis. Increased HOTAIR expression in breast cancer is transcriptionally induced by Estradiol [20]. PCGEM1 [21] and PCAT-1 [22] are prostate cancer associated non-coding RNA transcripts that are new components of cell apoptosis and proliferation pathways. The studies on lncRNAs are starting to become the center of esophageal cancer biology in the past two years. Wu et al found a long non-coding RNA transcript, AFAP1-AS1, highly expressed in esophageal adenocarcinoma, the functional experiments showed AFAP1-AS1 promotes invasion and metastasis in esophageal cancer cells [23]. More recently, aberration lncRNA expressions in esophageal cancer were reported, such as up-regulation of HOTAIR [19,24-26], taurine-upregulated gene 1 (TUG1) [27], PlncRNA1 [28], POU3f3 [29], FOXCUT [30], HNF1A-AS1 [31], ANRIL [32] and signature identification (lncRNAs ENST00000435885.1, XLOC_013014 and ENST00000547963.1) [33]. However, our understanding of lncRNAs in esophageal cancer biology is still in infancy.

In our previous study, in silico locus control analysis identified lncRNA ESCCAL-337 (chr3:171506370-171528740) and ESCCAL-356 (chr5:1544500-1567142, reverse strand) may modulate lipid metabolism genes contributing to esophageal cancer development [34]. In this report, we use more stringent bioinformatics analysis to dissect the ESCC-related lncRNAs and unbiasedly construct the interactions between lncRNAs and coding-gene expression. From this analysis, we choose a significantly up-regulated ESCCAL-1 lncRNA for further experimental investigation with small interfering RNA technique, the results suggest the potential role of ESCCAL-1 inhibits apoptosis and promote invasion.

## Methods

### Patient samples

Primary ESCC tumors and adjacent non-neoplastic tissues were obtained from four patients (all male, average age was 66 years old) with later clinical stages who underwent surgical treatment at Linxian Hospital on May 2012. The informed consent was obtained from the patients before surgery. The study protocol was approved by the Institutional Review Board for the use of human subjects at Zhengzhou Central Hospital. All tissues were frozen in liquid nitrogen immediately after surgical resection. None of the patients had prior chemotherapy or radiotherapy, nor did they have any other serious diseases. All ESCC tissues were histopathologically diagnosed by at least two independent senior pathologists.

#### Microarray hybridization

Total RNAs were extracted using Trizol reagent (Invitrogen, Carlsbad, CA, USA) following manufacturer’s instruction. The quality of RNAs was measured with a 2100 Bioanalyzer (Agilent technology, USA). Input of 100ng of total RNA was used to generate Cyanine-3 labeled cRNA according to the Agilent One-Color Microarray-Based Gene Expression Analysis Low for Input Quick Amp Labeling kit (v6.0). Samples were hybridized on Agilent SurePrint G3 Human GE 8x60K Microarray (Design ID 028004). Arrays were scanned with the Agilent DNA Microarray Scanner at a 3 μm scan resolution and data were processed with Agilent Feature Extraction 11.0.1.1. The microarray data discussed in this article have been deposited in National Center for Biotechnology Information (NCBI) Gene Expression Omnibus (GEO) and are accessible through (GEO) Series accession number GSE45350 (http://www.ncbi.nlm.nih.gov/geo/query/acc.cgi?acc=GSE45350). This data was also used in our previous study with different locus control analysis [34].

#### Bioinformatic analysis

The raw intensity data was exported to GeneSpring 12.0 (Agilent Technologies, Santa Clara, CA, USA) for quantile normalization. The normalized data containing 42544 probes were further analyzed using the R program. 139 positive control probes were removed. We then defined the coding (“NM_”, “XM_”) and non-coding (“lincRNA”, “NR_” and “XR_”) genes in the normalized data according to the definition of RefSeq accession format (http://www.ncbi.nlm.nih.gov/projects/RefSeq/key.html). The landscape of whole transcriptome (lncRNAs + coding RNAs), lncRNAs only and coding RNAs only were analyzed with gene expression dynamic inspector (GEDI) [34,35].

#### Differential expression

Differential expression analysis was conducted in R environment using Limma (linear models for microarray data) package [36]. Differentially expressed genes were considered with P-value of 0.001 with FDR control. DE-lncRNAs and DE-coding gene expressions are displayed in genome view (hg19) with RCircos plot.

#### Co-expression analysis

Co-expression analysis was conducted in R environment using RedeR package [37]. RedeR package has a strong statistical pipeline for several network analysis. Here, we have used co-expression analysis, computing a null distribution via permutation and returning the significant correlation values. We performed 1000 permutations to build the null distribution and used Pearson correlation. We considered correlations with p-value less than 0.01 with FDR adjustment as significant. The hierarchical cluster analysis used the *complete* method which considers the distances of each individual component to progressively computing the clusters, until find a stable state. The final result is a dendrogram presenting hierarchical leaves, which has been used to plot the network. To clear the visualization, clusters ware nested using the fourth level of dendrogram to build the nests.

#### Validation by quantitative real time polymerase chain reaction (Q-PCR)

Q-PCR analysis was performed on additional 26 matched ESCC and adjacent non-neoplastic tissues for selected lncRNAs. The primer sequences for PCR are as follows: HOTAIR, forward 5’-GGTAGAAAAAGCAACCACGAAGC-3’ and reverse 5’- ACATAAACCTCTGTCTGTGAGTGCC-3’; esophageal squamous cell carcinoma-associated lncRNA-1 (ESSCAL-1) (chr8:76121095-76189420 reverse strand), forward 5’-CCAGACAGCAGCAAAGCAAT-3’ and reverse 5’- GGAAGCAGCAAATGTGTCCAT-3’; GAPDH was used as a control, forward 5’-CCGGGAAACTGTGGCGTGATGG-3’ and reverse 5’-AGGTGGAGGAGTGGGTGTCGCTGTT-3’. The total RNA extraction with Trizol reagent (Invitrogen, Carlsbad, CA, USA) and cDNA preparation (High capacity cDNA reverse transcription kit, life technology, USA) were done following manusfactures’ instruction. Q-real time PCR was performed on ABI7900HT PCR machine (Applied Biosystems, USA) using faststart universal SYBR Green Master Mix kit (Roche, Germany). 1μl of cDNA for each sample in 10 μl reaction. The thermocycle conditions are as follows: initial denaturation at 94°C for 10 minutes, followed by 94°C for 15 seconds, 60°C for 1 minute, for 40 cycles. Ct (cycle of threshold) values were used to calculate the relative lncRNA expression level. ΔCt = (Ct _lncRNA_-Ct_GAPDH_), while ΔΔCt = (Ct _lncRNAinTumor_ -Ct_GAPDHinTumor_) - (Ct _lncRNAinNormal_ -Ct_GAPDHinNormal_). The fold change of lncRNA expression in ESCC relative to normal tissue is 2 ^–ΔΔCt^.

#### Long non-coding RNA ESCCAL-1 knockdown experiment

Three small interfering RNAs were designed to target various region of ESCCAL-1 gene expression. The sequences for ESCCAL-1 siRNA_1, 2, 3 are: 5’ CCGGGAGGAGAAAGACCCAAGCACTCGAGTTTGCTTGGGTCTTTCTCCTCTTTTTG 3’; 5’ CCGG CACATTCATGGTGTTGAGAAA CTCTAG TTTCTCAACACCATGAATGTG TTTTTG 3’; and 5’ CCGG TAGCAGAACAACACCTGGCAA CTCGAG TTGCCAGGTGTTGTTCTGCTA TTTTTG 3’, respectively. The negative control siRNA sequence is: 5’-CCGGTTCTCCGAACGTGTCACGTTTCAAGAGAACGTGACACGTTCGGAGAATTTTTG-3’. These oligonucleotides were annealed and subcloned into the Agel and EcoR1 sites of the hU6-MCS-CMV-EGFP (GV115) viral vector (GeneChem, shanghai) according to the manufacture’s instruction. The shuttle vector and viral packaging system were cotransfected into HEK293T cells to replicate competent lentivirus. The average titer is 1×10^8^ TU/ml. The esophageal cancer cell line EC9706 was used for viral infection. The infection efficiency was greater than 80% as monitored with GFP protein expression. After 72 h infection with various ESCCL-1 siRNAs, the cells were harvested and total RNA were extracted to examine ESCCAL-1 expression as described in Q-RT-PCR section.

#### Apoptosis and transwell assay

For apoptosis assay, the EC9706 cells were infected with ESCCL-1 siRNA-1, which has maximum knockdown effect, or negative control siRNA. Five-day post-infection, cells were collected and stained with an apoptotic marker, Annexin V (ebioscience 88-8007, USA) per manufacture’s manual then detected with flow cytometry (FACSCalibur, BD, USA).

For invasion assay, The EC9706 cells (1×10^5^ cells) were seeded into Transwell champer (corning 3422), then placed into wells containing media with 30% fetal bovine serum, and cultured for 24 hours, the cells migrated into membrane were stained with Giemsa, measured with OD570; meanwhile, 5x10^3^ cells were seeded into 96 well plate, and cell numbers were measured with MTT value (OD490), the invasion rate = OD570/OD490.

#### Statistical analysis

Each condition has three replicates, data present with mean and standard deviation. T-test was used for p value calculation. Significant difference is considered with p < 0.05.

## Results and discussion

### The landscape of ESCC transcriptome

**Figure 1:**
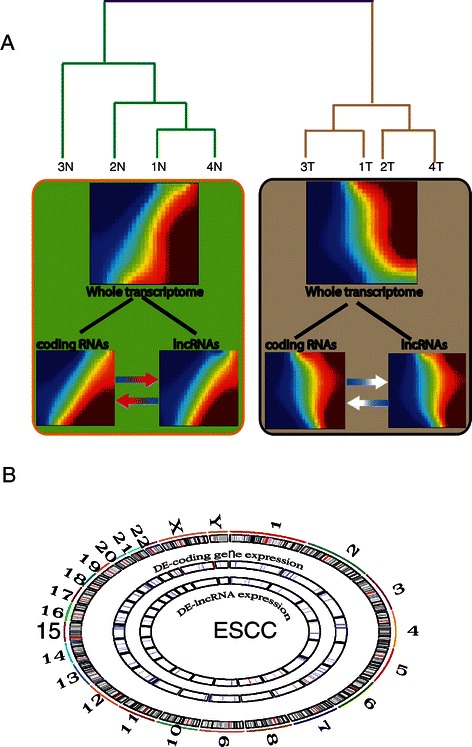
**Transcriptomic landscape of esophageal squamous cell carcinoma (ESCC). (A)** Unsupervised hierarchical clustering analysis of genome-wide RNA transcripts in ESCC and adjacent normal tissue using a microarray with 7,419 long noncoding RNAs (lncRNAs) and 27,958 coding RNAs. A self-organizing map (SOM) of either whole transcriptome (both lncRNAs and mRNAs) or lncRNAs or mRNA was produced from each group samples using gene expression dynamic inspector (GEDI). Mosaic patterns are pseudo-colored SOMs to show integrated biological entity in each sample. The color gradient from red to blue color indicates the expression level from high to low. **(B)** Liner models for microarray data analysis (limma) identified differentially expressed lncRNAs (DE-lncRNAs) and coding genes (DE-coding gene) in ESCC relative to normal tissue. FDR < =0.01, P <0.001. DE-lncRNAs and DE-coding gene expressions are displayed in genome view (hg19) with RCircos plot. In each circle view, blue line: downregulation, red line: upregulation.

Gene expression profiles of ESCC predominantly focused on mRNAs [38-40] and microRNAs [41,42] in the past years. These transcripts only represent small proportion of whole transcriptome in esophageal cancer genome. LncRNAs are emerging as a novel class of noncoding RNAs that are pervasively transcribed in the genome [1], their expression profiling in ESCC has not been well investigated systematically. In our study, the genome-wide transcriptome including 7,419 intergenic lncRNAs along with 27,958 mRNAs were examined in ESCC and adjacent normal tissue. The expression of whole transcriptome (lncRNAs and mRNAs) on the microarray is able to distinguish ESCC from normal tissue with unsupervised hierarchical clustering (Figure 1A). Next, we examined the whole transcriptomic pattern from each groups using gene expression dynamics inspector (GEDI), which accentuates a novel holistic perspective [35]. The mosaic pattern of whole transcriptome derived from normal tissues is different from that of ESCC. We further measured the holistic expression profiling of either lncRNAs or coding RNAs from each sample, again the overall changes from normal to cancer state were also seen in the components of whole transcriptome of ESCC. From the view of systems biology, neither lncRNAs nor mRNA expression pattern could represent the whole transcriptome of ESCC, the interaction between the components (lncRNAs and coding RNAs) may be reshaping the landscape of the whole transcriptome during ESCC development.

### Differential expression of lncRNAs and coding RNAs in ESCC

In order to find what coding and non-coding RNAs contribute to the cellular malignant transformation, we used liner model for microarray analysis (limma) to exam the expression level of RNA transcripts remarkably changed in ESCC relative to normal tissue. Limma model has more statistical power to detect differentially expressed genes, and also produces relative more reliable gene ranking with fewer false discoveries. From this analysis, a total of 128 genes including coding and non-coding (lncRNAs) were identified with highly significant change (p < 0.001, Bonferroni correction) (Additional file 1: Table S1). Among them, 95 are coding genes, 33 are noncoding genes. Most of these significantly differentially expressed RNA transcripts were downregulated in ESCC (Figure 1B). A subset of these differential gene expressions are consistent with the literature, such as aberrant coding gene expression of ARSF, DMRTA1, MAGEA1, ECM1, HIPK2, and PIK3C2G were detected in other studies in esophageal cancer [43,44]. Non-coding gene ESCCAL-1 has been validated in extra three ESCC samples as shown in our previous study [34].

### Construction of co-expression network between coding and lncRNA genes in ESCC

**Figure 2:**
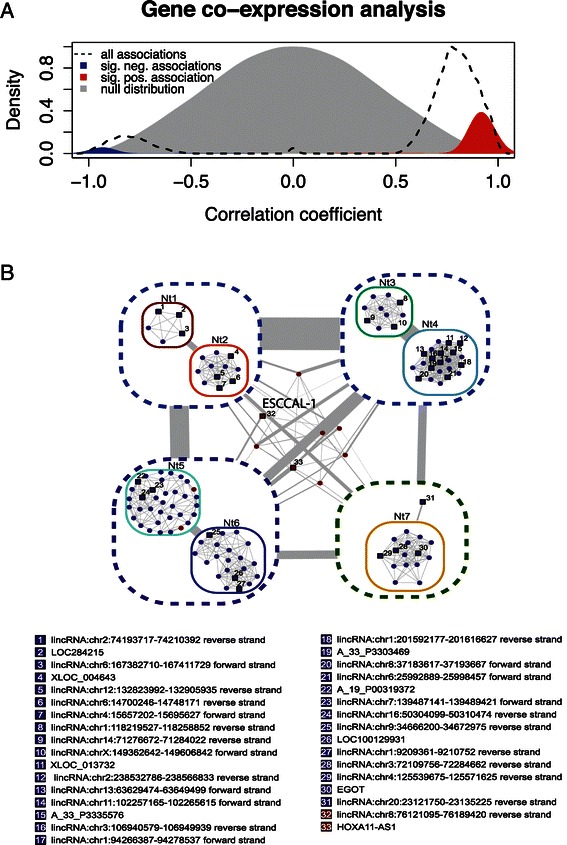
**Co-expression network of esophageal squamous cell carcinoma. (A)** Pearson correlation of DE-lncRNAs and DE-coding genes. **(B)** Co-expression of esophageal squamous cell carcinoma has been performed after differential expression analysis involving tumor (n = 4) against normal tissue (n = 4). Resulting clusters were nested to clear the visualization. The nests were numbered from Nt 1 to Nt7. Link width represents number of interactions among clusters. Red nodes represent increased transcription comparing to control and blue nodes represent decreased transcription comparing to control. Circles represents coding RNA and squares represents long non-coding RNA. Dashed lines enclosing clusters of co-expressed genes. Long non-coding RNAs are numbered and the respective annotation is present on the bottom of the figure. For the coding RNA annotation, please see Additional file 2: Figure S1.

The function of specific coding genes [38-40] and microRNAs [41,42] are well studied in ESCC, in contrast, lncRNA functions in ESCC remain largely unknown. The compelling evidence show that biological networks contain modules of genes or proteins that may function in the same pathway, genes or proteins inside a module can be co-regulated, they are often represented by one single node in the network [7]. Therefore, Computational construction of coding and non-coding gene co-expression network with different algorithms could infer the lncRNA potential biological functions [7,8]. We used a well-developed computational algorithm, RedeR [37], to construct the co-expression network on the 128 differentially expressed RNA transcripts (coding RNAs and lncRNAs). The distribution of association between coding genes and lncRNA gene is calculated (pearson correlation, p < 0.01) and displayed in Figure 2A. The significant correlated genes were selected for the construction of co-expression network. We dissected eight modules, which comprise of various nodes in the network. Interestingly, most modules include down-regulated genes, only one module is the connection of up-regulated genes (Figure 2B, for the coding RNA annotation, please see Additional file 2: Figure S1.). A grouped genes in each nodes may execute same or similar functions, which is biological basis for inference of unknown gene functions including lncRNAs.

### Prediction of lncRNA functions based on co-expression network

**Figure 3:**
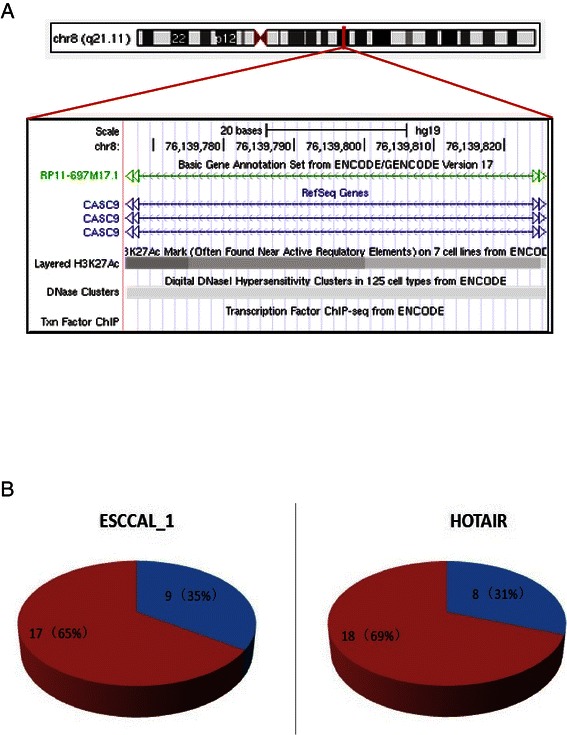
**The lncRNA ESCCAL-1 expression in ESCC. (A)** UCSC genome browser view of ESCCAL-1 (chr8:76139826-76139767). This lncRNA is also known as RP11-697M17.1 from GENCODE transcript annotation or ENST00000504531.2 or CASC9 (cancer susceptibility candidate 9) from Refseq annotation. The transcriptional regulation of ESCCAL-1/CASC9 has been evaluated by ENCODE (detailed seen Additional file 3: Figure S2). **(B)** ESCCAL-1 expression was further examined in independent 26 pair-matched normal and ESCC samples, its expression was significantly higher in 17 out of 26 (65%) ESCC samples relative to normal tissue. LncRNA HOTAIR, a known oncogenic lncRNA was also measured as a positive control.

Since lncRNAs’ functions are largely unknown, prediction of their putative functions now rely on the co-expression network [7,8]. From the co-expression network based on 128 differential expression of lncRNAs and coding genes, we focused on one of the up-regulated nodes in meddle of Figure 2B. This selected module is composed of two lncRNAs: one is HoxA11-AS1 (HoxA11 antisense RNA), another is ESCCAL-1(Esophageal Squamous Cell Carcinoma Associated LncRNA-1). The module also includes six protein-coding genes, they are COL10A1, MMP11, LEPREL4, FNDC1, MAGEA1, INHBA. HoxA11-AS1 is evolutionary conservation and tissue-specific expression [45], It was proposed that HOXA11 antisense represses HOXA11 expression by competing for transcription of the common gene, rather than by sense/antisense interaction [46]. The link of HOXA11-AS1 to cancer is unknown; it is the interest for future study. Because we have confirmed elevated expression of ESCCAL-1 in independent three samples [34], and ESCCAL-1 is the focus of this study. LncRNA ESCCAL-1 is also named RP11-697M17.1 from GENCODE transcript annotation ENST00000504531.2 or CASC9 (cancer susceptibility candidate 9) from Refseq annotation (Figure 3A). The transcriptional regulation of ESCCAL-1/CASC9 has been evaluated by ENCODE (detailed seen Additional file 3: Figure S2). For coding genes in this selected node, MMP11 [47], FNDC1(also known as MEL4B3) [48], MAGEA1 [49], INHBA [50] are experimentally demonstrated positive associated with cancers. According to this prediction, we reasoned the “oncogenic” role of ESCCAL-1 in ESCC development. Therefore, we examined ESCCAL-1 expression in 26 paired wise independent cohort samples using quantitative RT-PCR, it turns out that ESCCAL-1 is highly expressed in 65% of ESCC samples relative to adjacent normal tissues, its higher expression frequency in ESCC is compatible with onco-lncRNA HOTAIR expression in ESCC [19].

### Reverse the phenotype of esophageal cancer cells by reducing ESCCAL-1 expression

**Figure 4:**
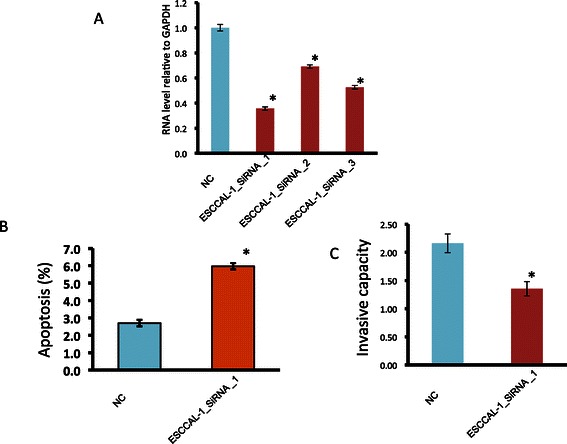
**Knockdown of ESCCAL-1 expression increases apoptosis and reduces invasion in vitro. (A)** ESCCAL-1 expression was significantly reduced by three individual small interfering RNAs with maximum ~65% reduction in EC9706 esophageal cancer cells infected with ESCCAL-1 siRNA_1. **(B)** Apoptosis assay showed EC9706 cells infected with ESCCAL-1_siRNA_1 increased as twice apoptotic cells as control cells. **(C)** Transwell invasion assay displayed reduced cell migration of ESCCAL-1_siRNA_1 infected EC9706 cells in comparison with control cells. * P < 0.05.

We want to know how esophageal cancer cell behavior changes if knocking down the expression of ESCCAL-1 lncRNA, we therefore designed three small interfering RNAs targeting various region of ESCCAL-1 sequencing. The esophageal cancer cell line EC9706 with higher ESCCAL-1 expression (data not shown) was infected with lentivirus containing either control or ESCCAL-1 siRNA, after 72 h infection, all three ESCCAL-1 siRNAs can remarkably reduce ESCCAL-1 expression with maximal inhibition of 65% (Figure 4A). We used ESCCAL-1 siRNA-1 for further biological experiments. The apoptotic cells increased and invasive capacity decreased significantly in cell infected with ESCCAL-1 siRNA-1 compared to control cells (p < 0.05). This experimental data suggest that ESCCL-1 may act together with abovementioned coding genes to inhibit cell death program and facilitate the invasion and metastasis. As we are revising our manuscript, at least 11 lncRNAs were reported to be upregulated in ESCC relative to adjacent normal tissue, overexpression of these lncRNAs are associated poor prognosis and metastasis of ESCC [19,23,27-33]. Core signaling pathways may be disrupted by these aberrant lncRNA expressions, such as WNT pathway is activated by HOTAIR over expression [25], TGFbeta1 pathway is inhibited by ANRIL [32]. Our study here provides an additional ESCC-associated lncRNA as a biomarker for ESCC diagnosis and prognosis. The detail mechanisms remain to be investigated.

## Conclusion

We performed a genome-wide survey of the expression of lncRNAs and coding RNAs from primary ESCC tissue and adjacent normal tissue in four individuals. The overall transcriptomic landscape is able to distinguish malignant from normal tissue in each patients. Interaction between lncRNAs and coding RNAs may reshape the landscape of the whole transcriptome during ESCC development. The stringent liner model analysis identified 128 significantly differentially expressed coding and non-coding RNA transcripts in ESCC relative to normal tissue. More strikingly, the co-expression network constructed the potential functional modules and sub-networks linking lncNRAs with coding genes. From one of the sub-networks, we inferred ESCCAL-1 putative oncogenic role in ESCC and verified its up-regulation in an independently cohort. The knockdown of ESCCAL-1 expression increases apoptosis and decrease invasion *in intro*. This proof of principle study provides a systematic strategy to study lncRNA functionality.

## Additional files

Additional file 1: Table S1. Differential expression of lncRNAs and mRNAs in esophageal squamous cell carcinoma relative to adjacent normal tissue (p < 0.001, FDR < 0.01).
Additional file 2: Figure S1. Co-expression of esophageal squamous cell carcinoma has been performed after differential expression analysis involving tumor (n=4) against normal tissue biopsies (n=4). Seven subnetworks were determined from the differentially expressed coding and lncRNA genes. Red nodes represent increased transcription comparing to control and blue nodes represent decreased transcription comparing to control. Circles represents coding RNA and squares represents long chain non-coding RNA. Long non-coding RNAs are numbered and the respective annotation is present on the bottom of the figure.
Additional file 3: Figure S2. LncRNA ESCCAL-1 (chr8:76139826-76139767) was displayed in UCSC genome browser with transcriptional and epigenetic modification detected by ENCODE analysis.

## Abbreviation

ESCC: Esophageal squamous cell carcinoma
AFAP1: Actin filament associated protein
AFAP1-AS1: AFAP1 antisense RNA
lncRNA: Long noncoding RNA
ESCCAL: ESCC-associated lncRNA
MALAT-1: Metastasis-associated in lung adenocarcinoma transcript 1
HOTAIR: HOX transcript antisense RNA
PCAT-1: Prostate cancer associated noncoding RNA transcript1
PCR: Polymerase chain reaction
Xist: X inactivative specific transcript
