# Supplementary material for: Prediction of long noncoding RNA functions with co-expression network in esophageal squamous cell carcinoma
Source: BMC Cancer. 2015 Mar 24;15:168. doi: 10.1186/s12885-015-1179-z (PMC4377028; doi:10.1186/s12885-015-1179-z)
Supplement: Additional file 2: Figure S1. — Co-expression of esophageal squamous cell carcinoma has been performed after differential expression analysis involving tumor (n=4) against normal tissue biopsies (n=4). Seven subnetworks were determined from the differentially expressed coding and lncRNA genes. Red nodes represent increased transcription comparing to control and blue nodes represent decreased transcription comparing to control. Circles represents coding RNA and squares represents long chain non-coding RNA. Long non-coding RNAs are numbered and the respective annotation is present on the bottom of the figure. [file 12885_2015_1179_MOESM2_ESM.pdf]

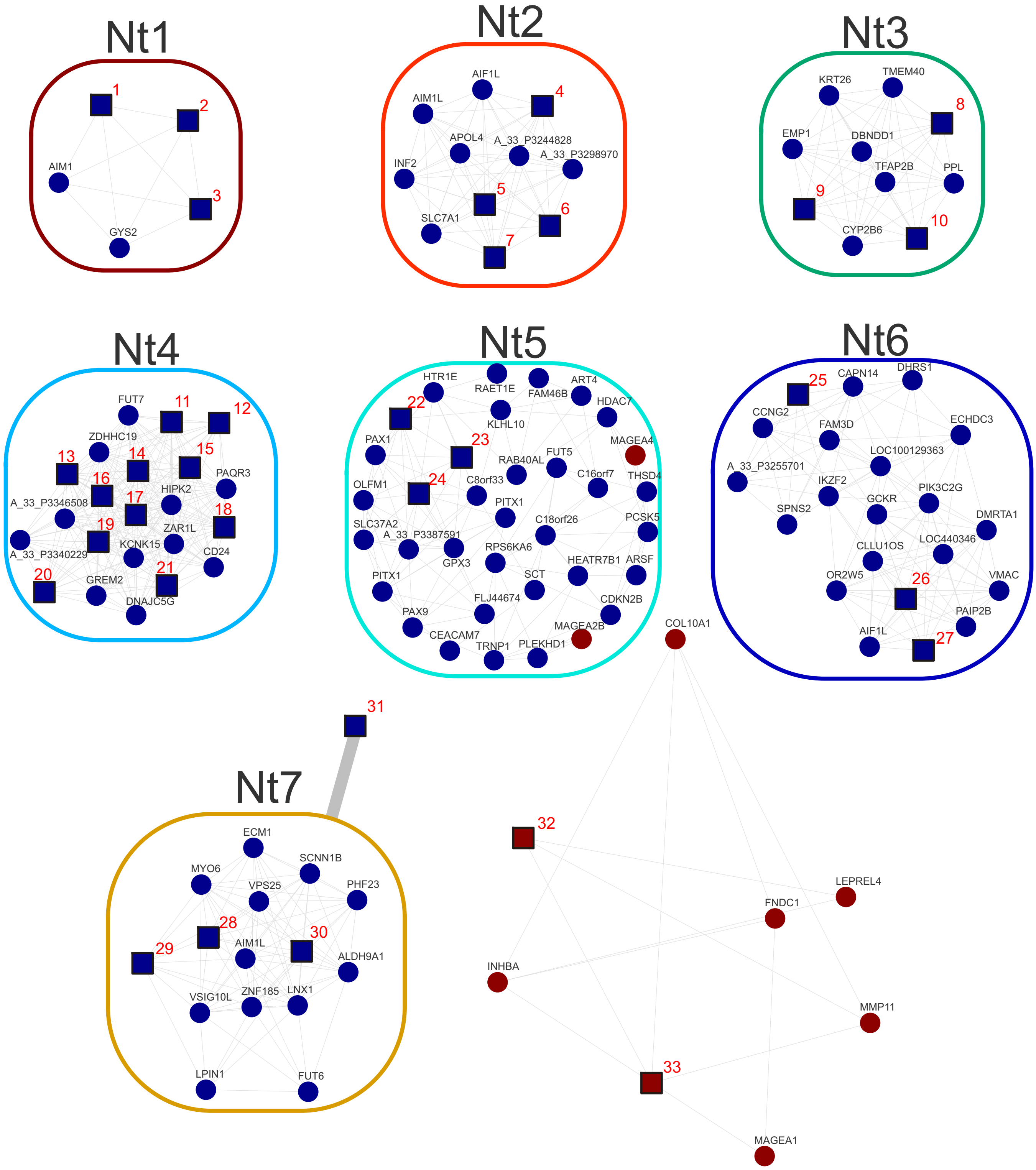

1 lincRNA:chr2:74193717-74210392 reverse strand
2 LOC284215
3 lincRNA:chr6:167382710-167411729 forward strand
4 XLOC_004643
5 lincRNA:chr12:132823992-132905935 reverse strand
6 lincRNA:chr6:14700246-14748171 reverse strand
7 lincRNA:chr4:15657202-15695627 forward strand
8 lincRNA:chr1:118219527-118258852 reverse strand
9 lincRNA:chr14:71276672-71284022 reverse strand
10 lincRNA:chrX:149362642-149606842 forward strand
11 XLOC_013732
12 lincRNA:chr2:238532786-238566833 reverse strand
13 lincRNA:chr13:63629474-63649499 forward strand
14 lincRNA:chr11:102257165-102265615 forward strand
15 A_33_P3335576
16 lincRNA:chr3:106940579-106949939 reverse strand
17 lincRNA:chr1:94266387-94278537 forward strand
18 lincRNA:chr1:201592177-201616627 reverse strand
19 A_33_P3303469
20 lincRNA:chr8:37183617-37193667 forward strand
21 lincRNA:chr6:25992889-25998457 forward strand
22 A_19_P00319372
23 lincRNA:chr7:139487141-139489421 forward strand
24 lincRNA:chr16:50304099-50310474 reverse strand
25 lincRNA:chr9:34666200-34672975 reverse strand
26 LOC100129931
27 lincRNA:chr1:9209361-9210752 reverse strand
28 lincRNA:chr3:72109756-72284662 reverse strand
29 lincRNA:chr4:125539675-125571625 reverse strand
30 EGOT
31 lincRNA:chr20:23121750-23135225 reverse strand
32 lincRNA:chr8:76121095-76189420 reverse strand
33 HOXA11-AS1
