# Supplementary figures and images for: Prediction of long noncoding RNA functions with co-expression network in esophageal squamous cell carcinoma
Source: BMC Cancer. 2015 Mar 24;15:168. doi: 10.1186/s12885-015-1179-z (PMC4377028; doi:10.1186/s12885-015-1179-z)

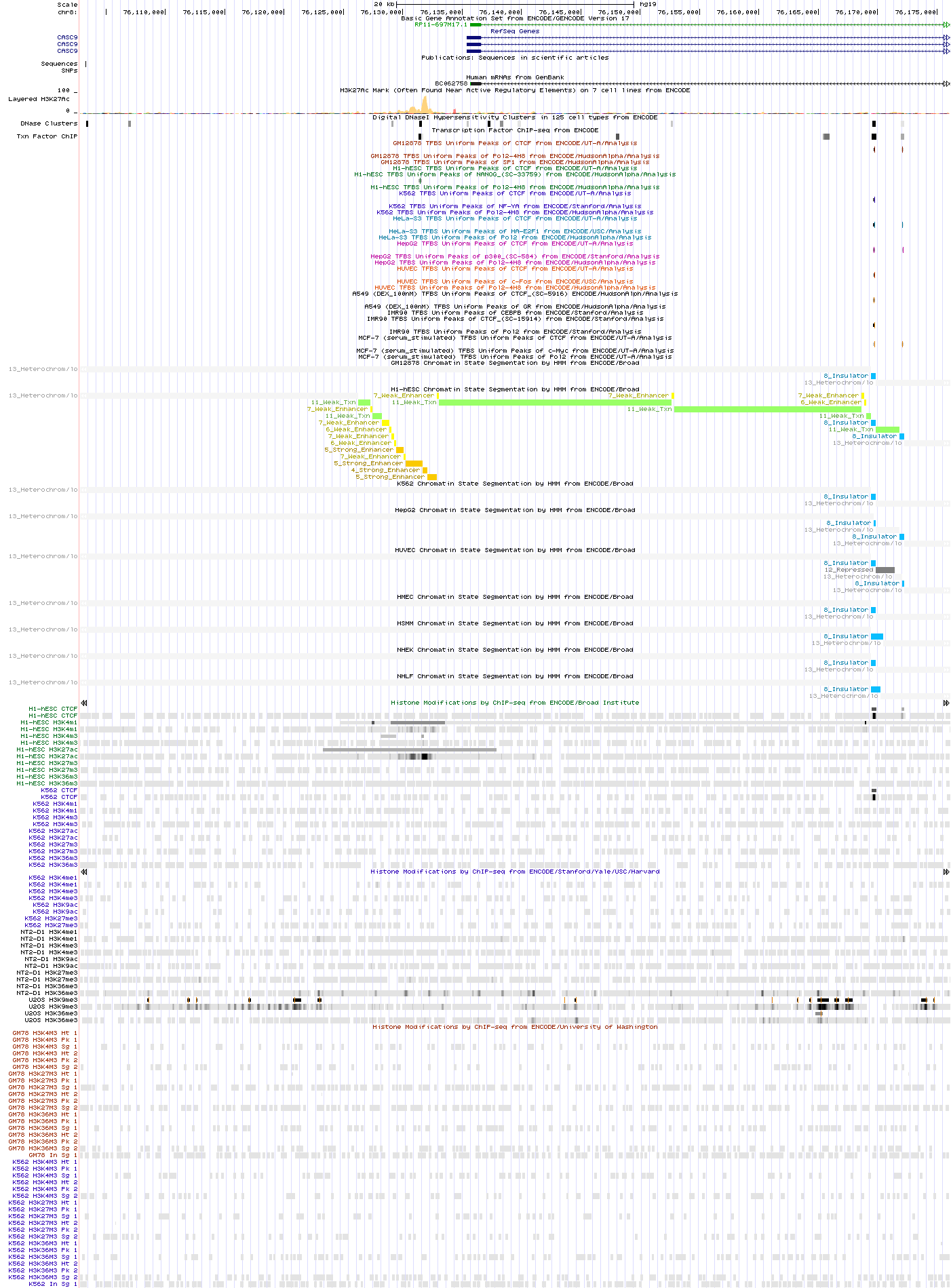

Supplement: Additional file 3: Figure S2. — LncRNA ESCCAL-1 (chr8:76139826-76139767) was displayed in UCSC genome browser with transcriptional and epigenetic modification detected by ENCODE analysis. [file 12885_2015_1179_MOESM3_ESM.png]
